# Combined Effect of Metformin and miR-145/miR-23b Co-Transfection on Proliferation and Progression in 2D and 3D Epithelial Ovarian Cancer Models

**DOI:** 10.3390/cells15100933

**Published:** 2026-05-19

**Authors:** Matías Alfonso Rubio, Eduardo Velásquez, Sofia Antonucci, María José Sánchez, Carmen Romero

**Affiliations:** 1 Laboratory of Endocrinology and Reproductive Biology, Clinical Hospital University of Chile, Santiago 8380456, Chile; 2 Department of Obstetrics and Gynecology, Clinical Hospital, Faculty of Medicine, University of Chile, Santiago 8380456, Chile; 3 Department of Biochemistry and Molecular Biology, Faculty of Chemistry and Pharmaceutical Sciences, University of Chile, Santiago 8380493, Chile

**Keywords:** miR-145, miR-23b, epithelial ovarian cancer, VEGF, spheroids, 3D cancer model

## Abstract

Epithelial ovarian cancer (EOC) remains a lethal malignancy requiring novel therapeutic strategies due to high recurrence and chemoresistance. This study evaluated the combined antitumor effect of metformin and the co-transfection of tumor-suppressor microRNAs miR-145 and miR-23b in A2780 and OV90 EOC cell lines using both 2D and 3D models. In monolayer cultures, our approach significantly reduced the expression of proliferation markers Ki-67 and c-MYC, and decreased cell migration and invasion in both cell lines compared to controls. In 3D spheroid models, the treatment reduced VEGF secretion and relative spheroid area in A2780 cells, significantly increasing cytotoxicity; however, OV90 spheroids exhibited marked resistance. Fluorescent miRNA tracking revealed that this resistance occurs despite successful intracellular delivery, indicating an intrinsic biological resistance conferred by the 3D microenvironment. Overall, these findings suggest that the combined administration of metformin and miRs effectively limits tumor progression, but also strongly underscore the importance of using complex 3D models to accurately evaluate therapeutic efficacy and intrinsic resistance mechanisms.

## 1. Introduction

Epithelial ovarian cancer (EOC) stands as one of the most lethal gynecological malignancies globally. This disease is characterized by an asymptomatic clinical presentation in early stages and is frequently diagnosed in late stages. Worldwide, EOC ranks as the eighth most common cancer in women, accounting for approximately 3.7% of cases and 4.7% of cancer deaths in 2020 [1]. Patients with invasive EOC have a 5-year relative survival rate of only 32%, in contrast to the 92% observed in localized cases [2]. This disease can be classified into two groups: Type I tumors, which are clinically indolent and genetically stable, and Type II tumors, which are biologically aggressive and genetically unstable, thus being responsible for the majority of deaths [3,4]. Despite the advances in EOC research, the prognosis remains unfavorable due to recurrence and drug resistance, underscoring the need to optimize therapeutic strategies.

The standard treatment for advanced EOC consists of cytoreductive surgery followed by adjuvant chemotherapy based on platinum compounds and taxanes, such as paclitaxel [4,5]. However, although most patients initially respond, approximately 80% experience relapse due to the development of chemoresistance [5,6]. In recent years, targeted therapies have included monoclonal antibodies against vascular endothelial growth factor (VEGF) and poly (ADP-ribose) polymerase (PARP) inhibitors, particularly in patients with mutations in BRCA1/2 genes or homologous recombination deficiency [4]. Current research has shifted towards identifying emerging molecules and complementary therapies that may improve the efficacy of current treatments.

In this context, microRNAs (miRNAs) have been emerging as critical regulators in the disease and progression of EOC. miRNAs are small non-coding RNAs that regulate gene expression at a post-transcriptional level by degrading mRNA or inhibiting its translation [5]. Our group’s prior research indicated that nerve growth factor (NGF) is linked to reduced miR-145-5p levels [7]. This suggests a potential mechanism by which signals from the tumor microenvironment may suppress anti-tumor miRNAs. In this context, this suppression in EOC cell lines and tissues later deregulates key cellular processes, including cell proliferation, migration, and invasion [7]. Restoring miRNAs, such as miR-145 and miR-23b, can inhibit tumor progression by negatively regulating oncogenes and proteins associated with angiogenesis and drug resistance, including VEGF, c-MYC, ZEB1, and ABCB1 [8]. Novel strategies, such as the use of peptide-targeted gold nanoplatforms for the specific delivery of miR-145, have been shown to induce significant antitumor effects in EOC cells, demonstrating the therapeutic potential of these molecules [9]. Thus, the role of miRNAs in modulating angiogenesis and chemotherapy response reinforces their relevance as therapeutic targets [8,9].

Alternatively, metformin, a first-line antidiabetic drug, has accumulated great interest due to its antitumor properties. The use of this drug is associated with lower incidence and better survival in patients with EOC, particularly women with type 2 diabetes [10,11,12]. Evidence indicates that metformin inhibits the proliferation, migration, and invasion of EOC cells in vitro, as well as angiogenesis and metastasis formation in vivo [13]. One mechanism of this drug involves activating AMP-activated protein kinase (AMPK), thereby inhibiting the mTOR pathway, altering cellular metabolism, and reducing protein synthesis [14]. Furthermore, metformin can prevent the proliferative and proangiogenic effects induced by NGF in EOC cells and endothelial cells [15]. It has also been reported that metformin reduces the levels of NGF-induced tumor-promoting proteins, such as c-MYC and VEGF, and increases the expression of tumor suppressor miRNAs miR-145 and miR-23b [16]. Additional studies suggest that metformin sensitizes EOC cells to paclitaxel, overcoming chemoresistance by regulating metabolic pathways and inhibiting efflux transporters [6]. In addition, in phase II clinical trials, metformin has been shown to reduce the population of cancer stem cells (CSCs), which could positively impact recurrence-free survival [17].

To evaluate the efficacy of these new therapeutic strategies, selecting appropriate study models is fundamental. Traditional two-dimensional (2D) cell culture models have been the basis of cancer research but present significant limitations, such as the lack of cell–cell and cell–extracellular matrix interactions, and an inability to recapitulate in vivo tumor architecture and heterogeneity [18,19]. These deficiencies contribute to the high failure rate of drugs in clinical trials, as 2D models often do not accurately predict therapeutic response [20,21]. In contrast, three-dimensional (3D) models, such as spheroids and organoids, offer a more faithful representation of the tumor microenvironment, allowing the formation of nutrient and oxygen gradients and better mimicking drug resistance observed in patients [20]. These in vitro 3D models serve as a crucial bridge between 2D cultures and in vivo studies, providing a more accessible and controlled platform for preclinical evaluation [18,19,22].

Given the need to improve current therapies and the evidence supporting the individual potential of miRNAs and metformin, the next step is to test whether combining these compounds could yield better results. Given that metformin regulates the expression of miRNAs [16], and that the co-transfection of miR-145 and miR-23b has been shown to decrease proliferation and invasion [8], evaluating a complementary strategy is logical. Therefore, our objective is to evaluate the effect of a combined treatment involving the co-transfection of miRs and the use of metformin in a 2D and 3D EOC model, in order to determine its impact on viability and tumor progression within an environment that better mimics in vivo tissue complexity.

## 2. Methodology

### 2.1. EOC Cell Lines

A2780 and OV90 EOC cell lines were used. The A2780 cells originate from a human patient with primary EOC without chemotherapy treatment, and were obtained from the European Collection of Authenticated Cell Cultures (Salisbur, UK). OV90 cells are high-grade human EOC cell lines derived from metastases: these two cell lines were obtained from the American Type Culture Collection Virginia, Manassas, VA, USA.

### 2.2. 2D Cell Culture

A2780 and OV90 cells were cultured in RPMI 1640 medium (Gibco; Thermo Fischer Scientific, Inc., Waltham, MA, USA) supplemented with 10% treated fetal bovine serum (FBS; HyClone; Cytiva, Marlborough, MA, USA) at 37 °C with 5% CO_2_. Once the cells reached 80% confluence, they were plated for transfection. Cells stimulated with metformin (Sigma-Aldrich Co., Burlington, MA, USA) were in a final concentration of 0.5 mM for 48 h.

### 2.3. 2D Transient Transfection

Transient transfection was performed as described in [8].

Cells were transfected with miR-145 and miR-23b mimics: at a final concentration of 60 nM (Integrated DNA Technologies, Inc., Coralville, IA, USA). The transfection was performed using ViaFect transfection reagent (Promega Corporation, E498A., Madison, WI, USA) following the manufacturer’s protocol. Transfection was carried out at 37 °C for 48 h in RPMI 1640 medium supplemented with 2% FBS. For a negative control (Scramble), cells were transfected with a Scrambled Negative Control DsiRNA (Integrated DNA Technologies, 51-01-19-08) at a final concentration of 10 nM under the same conditions. Control cells were maintained under identical culture conditions (37 °C for 48 h in RPMI with 2% FBS) but without the transfection reagent or oligonucleotides. Also, we added a ViaFect group treated exclusively with the transfection reagent to account for vehicle toxicity.

### 2.4. 3D Cell Culture

A2780 and OV90 cells were cultured in DMEM/F12 supplemented with 10% FBS. Ultra-low attachment 96-well plates were used (Corning, #4520, New York, NY, USA). A total of 6000 cells were seeded and incubated for 4 days at 37 °C and 5% CO_2_ until the spheroids reached approximately 500 µm in diameter.

### 2.5. Transient Co-Transfection in Spheroids

Spheroids were transiently co-transfected after seeding 6000 cells per well in ultra-low attachment 96-well plates (Corning, #4520), following the previously described conditions. The co-transfection mixtures included a mixture of miR-23b and miR-145 (Integrated DNA Technologies), each at a final concentration of 240 nM, and a mixture of miR-23b and miR-145 (240 nM each) combined with metformin at a final concentration of 1 mM. The mixture of both miRNA are the same that were used in 2D cultures that are described in Table 1.

The co-transfection was performed using ViaFect transfection reagent (Promega, #E498A) according to the manufacturer’s protocol and lasted for 48 h in DMEM/F12 medium supplemented with 2% FBS.

The negative control involved transfecting cells with Scrambled Negative Control DsiRNA (51-01-19-08, Integrated DNA Technologies) at a final concentration of 10 nM under the same conditions. For the metformin condition, spheroids were treated with metformin at a final concentration of 1 mM in DMEM/F12 with 2% FBS. Control condition cells were simply maintained at 37 °C for 48 h in DMEM/F12 supplemented with 2% FBS.

Following the 48-h co-transfection period, spheroid size, viability, and cell count were assessed.

### 2.6. Functional Validation of miRNA Delivery Using Fluorescent Mimics

To evaluate the delivery efficiency of miRNAs into 3D spheroids, A2780 and OV90 spheroids were co-transfected using fluorescently labeled mimics: miR-145 conjugated with Cy5 (red signal) and miR-23b conjugated with Alexa 488 (green signal) (Integrated DNA Technologies). The transfection was performed maintaining the 240 nM concentrations for each mimic and using the ViaFect transfection reagent (Promega Corporation, E498A). After 4 h of co-transfection with DMEM/F12 0% FBS media, the spheroids are washed three times with PBS. Then, whole-spheroid fluorescence imaging was performed to assess intracellular localization and penetration. Images were acquired using a Cytation 5 cell imaging multimode reader (BioTek., Winooski, VT, USA).

### 2.7. Immunocytochemistry (ICC)

For monolayer culture ICC assays, 70,000 cells were seeded in 24-well plates containing a 12 mm round coverslip in RPMI medium with 10% FBS. Cells were allowed to grow for 24 h; they were then fixed with 4% paraformaldehyde (PFA) for 15 min. Washes with 1× PBS were then performed, followed by incubation with 0.3% Triton X-100 for 10 min. Endogenous peroxidase was blocked with 3% hydrogen peroxide for 15 min, protected from light, followed by blocking with 2% PBS/BSA for 5 min.

After this time, samples were incubated overnight at 4 °C with the primary antibody anti-Ki-67 (1:200; SC-23900, Santa Cruz, Dallas, TX, USA) or c-MYC (1:1000; #5605, Cell Signaling Technology, Danvers, MA, USA). Next day, samples were incubated with the secondary antibody anti-mouse 1:300 for Ki-67 (115-035-003, Jackson ImmunoResearch, West Grove, PA, USA) and anti-rabbit 1:1500 for c-MYC (111-035-003, Jackson ImmunoResearch) for 1 h at 37 °C. Subsequently, cells were washed and incubated with a 3,3′-Diaminobenzidine (DAB) solution (#K3468, Agilent Dako, Agilent Technologies., Santa Clara, CA, USA) for 3 min for Ki-67 and 1 min for c-MYC, followed by counterstaining with Harris hematoxylin (Merck Millipore. Burlington, MA, USA) for 40 s.

Cell dehydration was then performed using an alcohol series: 70% and 95% ethanol for 3 min, followed by 100% ethanol (Merck) for 3 min, and Histoclear (Fermelo Biotec. Santiago, Chile) for 5 and 10 min. Coverslips were then mounted using Entellan (#107961, Merck Millipore). 6–8 images were taken per insert using an optical microscope (Olympus BX51TF. Westborough, MA, USA). Images were analyzed using Image-Pro Plus 6.2 software (Media Cybernetics Inc., Silver Spring, MD, USA).

### 2.8. Cell Migration Assay

The cell migration assay was performed as described in [8] with minor modifications.

A total of 1,000,000 cells were seeded in 6-well plates to evaluate the effect of miR-145 and miR-23b co-transfection on the migration of EOC cells. Cells were transfected for 48 h. After the transfection period, migration was evaluated using a Costar^®^ Transwell permeable support (Corning, Inc.), which was coated on the bottom with 0.003 mg/mL fibronectin for A2780 cells or 0.01 mg/mL fibronectin for OV90 cells. A total of 45,000 EOC cells transfected with miRs and previously resuspended in RPMI without FBS were introduced into the upper chamber of the inserts. The inserts were placed in RPMI medium with 10% FBS, serving as a cell attractant. A2780 cells were allowed to migrate at 37 °C for 18 h, whereas OV90 cells were allowed to migrate at 37 °C for 24 h. Cells that migrated were fixed and stained with 0.1% crystal violet in 20% methanol for 1 h at room temperature. Cells that crossed the insert and remained attached to the lower membrane surface were observed under a light microscope (Olympus BX51TF; Olympus Corporation. Center Valley, PA, USA), and 6–8 images for each experimental condition were obtained. The cells in each image were counted using the Fiji ImageJ 1.54p software.

### 2.9. Cell Invasion Assay

The cell invasion assay was performed as described in [8], with minor modifications.

Cell invasion was assessed using the commercial BioCoat^®^ kit (Corning, Inc.) with Matrigel-coated Transwell inserts. Seeding and transfection procedure follows the same protocol as 2.8. Before use, the Matrigel inserts were hydrated for 2 h at 37 °C with RPMI medium without FBS. The inserts were then placed in a 24-well plate containing RPMI medium with 10% FBS, which served as a chemoattractant. To establish a chemoattractant gradient, 75,000 transfected cells were resuspended in serum-free RPMI medium and seeded into the upper chamber of the insert. The invasion assay was conducted for 24 h at 37 °C for both cell lines. Invading cells that reached the lower surface of the membrane were fixed in methanol at −20 °C for 2 min and stained overnight at room temperature with 1% toluidine blue. The inserts were examined under a light microscope (Olympus BX51TF; Olympus Corporation), and 6–8 images were captured for each experimental condition. Images of the attached cells were analyzed using the Fiji ImageJ program.

### 2.10. Cell Viability Assay

Cell viability in spheroids was assessed using the LIVE/DEAD Viability/Cytotoxicity Kit (Invitrogen, L3224. Carlsbad, CA, USA), which contains calcein-AM (Ca-AM) and Ethidium homodimer-1 (EthD-1) to identify live and dead cells, respectively. Seeding of 6000 cells was performed in an ultra-low attachment 96-well plate with a clear bottom and dark walls (Corning, #4520). After 48 h of co-transfection, incubation was carried out for 45 min with Ca-AM and EthD-1 at final well concentrations of 2 µM and 4 µM, respectively. Image acquisition was performed using the Cytation 5 cell image multimode reader with GEN5 software. Fluorescence IOD was determined using Image-Pro Plus 6.0 software (Media Cybernetics Inc., Rockville, MD, USA).

### 2.11. Spheroid Cell Count and Area Measurement

Following the 3D co-transfection described previously, 200 µL of culture medium was removed from each well, and each spheroid was disaggregated using 0.25% Trypsin-EDTA for 5 min at 37 °C; mechanical disaggregation was also performed prior to counting. Subsequently, a 10 µL aliquot was used for cell counting on the automated LUNA system (Logos Biosystems, Gunpo, Republic of Korea). For area measurement, photographs of the spheroids were taken under an optical microscope (Olympus CKX41. Center Valley, PA, USA) for the different conditions; using these images, the spheroid areas were determined in ImageJ.

### 2.12. VEGF Measurement by ELISA

VEGF levels were evaluated using the Quantikine Human VEGF Immunoassay kit (DVE00, R&D Systems. Minneapolis, MN, USA), which identifies VEGF 121 and VEGF 165 isoforms. For this assay, culture media from 2D and 3D cultures co-transfected with metformin were collected. The assay was performed following the manufacturer’s instructions, and absorbance was measured at 450 nm using a spectrophotometer (EL800 BioTek., Winooski, VT, USA).

### 2.13. Statistics

All experiments were conducted at *n* = 3 independent biological replicates. Results were analyzed using GraphPad Prism 9.0.0 software. Considering the results presented a nonparametric distribution, the Kruskal–Wallis test was used to compare the different conditions, followed by Dunn’s post hoc test. Results were considered significant at *p* < 0.05 and are expressed as mean ± standard error of the mean (SEM).

## 3. Results

### 3.1. Transient Co-Transfection of miR-145 and miR-23b in Combination with Metformin Decreases Proliferation in EOC Cells

**Figure 1:**
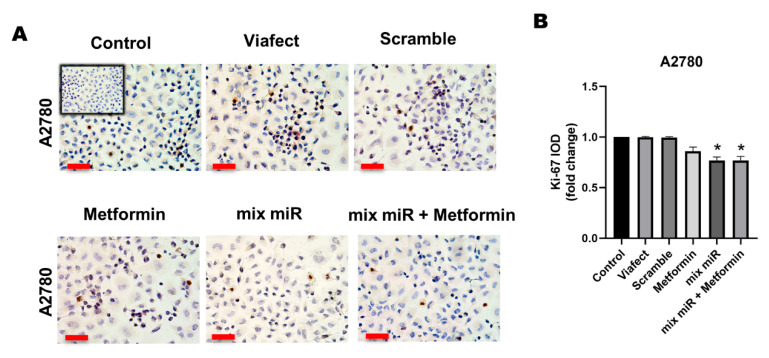

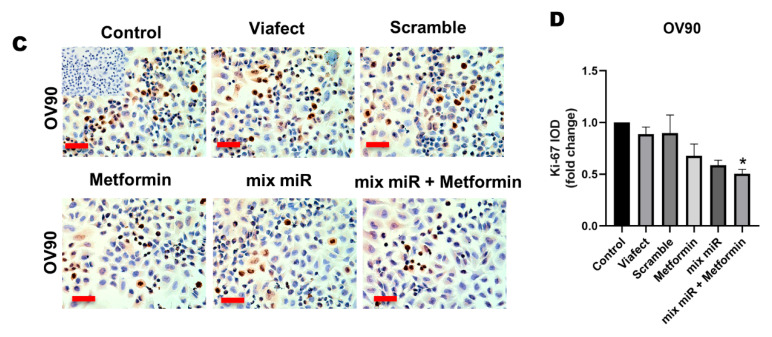
Effect of metformin plus a mixture of miRs on proliferation marker Ki-67 in EOC cells. Cell lines A2780 and OV90 were treated with vehicle (ViaFect), negative control (scramble), metformin, a mixture of miRs, and metformin plus the mixture of miRs for 48 h. (**A**) Representative images of specific immunocytochemistry for Ki-67 in A2780 cells. (**B**) Semi-quantification of Ki-67 positive cells. The panels represent the different conditions, which were normalized to the control condition and presented as fold change. (**C**) Representative images of specific immunocytochemistry for Ki-67 in OV90 cells. (**D**) Semi-quantification of Ki-67 positive cells. The graph is represented in the same fashion as (**B**). Images were obtained using 400× magnification. Scale bars represent 50 μm. *n* = 3 independent experiments. * = *p* < 0.05 according to the Kruskal–Wallis test followed by Dunn’s post-test. Results are expressed as mean ± standard error of the mean (SEM). Conditions are as following: Metformin: 0.5 mM metformin hydrochloride; mix miR: mixture of miRNAs (miR-23b and miR-145 at 60 nM each); mix miR + Metformin: 0.5 mM metformin plus the mixture of miR; mixture of miRNAs (miR-23b and miR-145 at 60 nM each).

**Figure 2:**
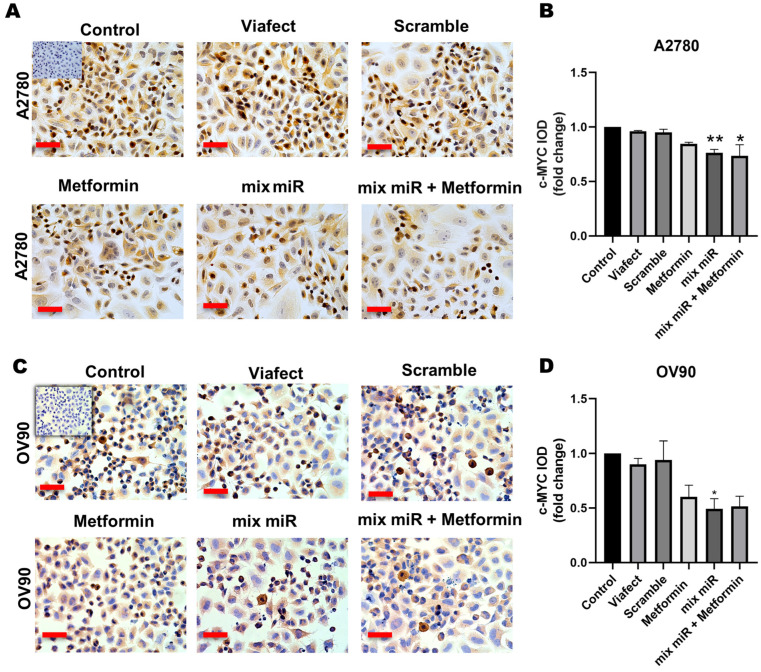
Effect of metformin plus a mixture of miRs on c-MYC protein in EOC cells. Cell lines A2780 and OV90 were treated with vehicle (ViaFect), negative control (scramble), metformin, a mixture of miRs, and metformin plus the mixture of miRs for 48 h. (**A**) Representative images of specific immunocytochemistry for c-MYC in A2780 cells. (**B**) Semi-quantification of c-MYC positive cells. The panels represent the different conditions, which were normalized to the control condition and presented as fold change. (**C**) Representative images of specific immunocytochemistry for c-MYC in OV90 cells. (**D**) Semi-quantification of c-MYC positive cells. The graph is represented in the same fashion as (**B**). Images were obtained using 400× magnification. Scale bars represent 50 μm. *n* = 3 independent experiments. * = *p* < 0.05, ** = *p* < 0.01 according to the Kruskal–Wallis test followed by Dunn’s post-test. Results are expressed as mean ± standard error of the mean (SEM). Conditions are as following: Metformin: 0.5 mM metformin hydrochloride; mix miR: mixture of miRNAs (miR-23b and miR-145 at 60 nM each); mix miR + Metformin: 0.5 mM metformin plus the mixture of miR; mixture of miRNAs (miR-23b and miR-145 at 60 nM each).

Immunocytochemical analysis showed that the treatments significantly reduced the expression of proliferation markers compared to the control condition. In A2780 cells, the combined therapy decreased Ki-67 levels and c-MYC expression significantly compared to the control group (Figure 1B and Figure 2B). In OV90 cells, significant reductions were also observed for Ki-67 (Figure 1D) and c-MYC (Figure 2D). However, direct statistical comparisons between the treatment groups revealed that the combined administration did not induce a significantly greater reduction in these specific proliferation markers than the individual treatments (metformin alone or miR mix alone). These findings indicate that while the therapy has a clear antiproliferative effect through the downregulation of key cell cycle proteins, the combination yields a complementary rather than synergistic effect on these specific targets under 2D conditions.

### 3.2. Transient Co-Transfection of miR-145 and miR-23b and Combined Therapy Decreases Cell Migration in EOC Cells

**Figure 3:**
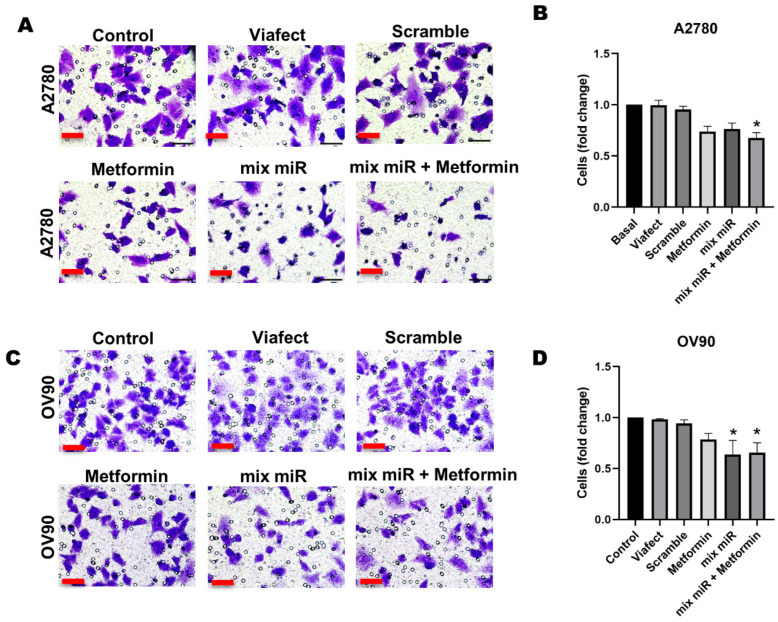
Metformin, in combination with a miR mixture, decreases cell migration in EOC cells. Cells were treated with 0.5 mM metformin, a mixture of miRs, and metformin plus the mixture of miRs for 48 h. After treatment, cells were seeded in transwell inserts and allowed to migrate for 18 h for A2780 cells and 24 h for OV90 cells. (**A**) Representative images of cell migration post-treatment in (**A**) for A2780 cells and (**C**) for OV90 cells. Semi-quantification of the number of migrated cells for A2780 cells (**B**) and OV90 cells (**D**). The panels represent the different conditions, which were normalized to Control condition and presented as fold change. Images were obtained using 400× magnification. Scale bars represent 50 μm. *n* = 3 independent experiments. * = *p* < 0.05 according to the Kruskal–Wallis test followed by Dunn’s post-test. Results are expressed as mean ± standard error of the mean (SEM). Conditions are as following: Metformin: 0.5 mM metformin hydrochloride; mix miR: mixture of miRNAs (miR-23b and miR-145 at 60 nM each); mix miR + Metformin: 0.5 mM metformin plus the mixture of miRNAs.

The cell migration assay showed that the treatments limit motility in both cell lines. In A2780, co-transfection and metformin together induced a significant 32% reduction in the number of migrated cells (Figure 3B). Meanwhile, in the OV90 line, the inhibitory effect was significant for both co-transfection and the combined therapy, with reductions of 36% and 34%, respectively, compared to the control (Figure 3D). These data suggest that administering these molecules effectively reduces the in vitro migratory potential of EOC cells.

### 3.3. Transient Co-Transfection of miR-145 and miR-23b in Combination with Metformin Decreases Cell Invasion in EOC Cells

**Figure 4:**
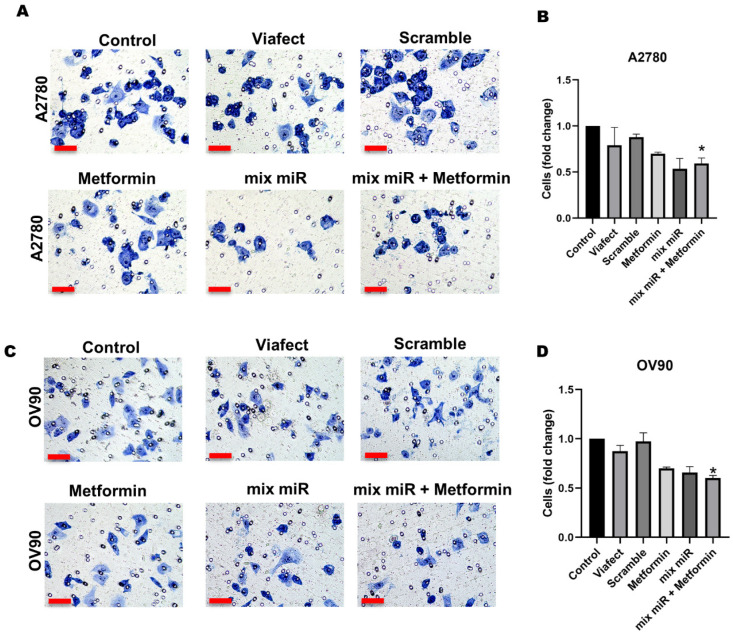
Metformin, in combination with a miR mixture, decreases cell invasion in EOC cells. Cells were treated with 0.5 mM metformin, a mixture of miRs, and metformin plus the mixture of miRs for 48 h. After treatment, cells were seeded in transwell inserts coated with Matrigel and allowed to invade for 24 h. (**A**) Representative images of cell invasion post-treatment in (**A**) for A2780 cells and OV90 cells (**C**). Semi-quantification of the number of invaded cells for A2780 cells (**B**) and OV90 cells (**D**). The panels represent the different conditions, which were normalized to Control condition and presented as fold change. Images were obtained using 400× magnification. Scale bars represent 50 μm. *n* = 3 independent experiments. * = *p* < 0.05 according to the Kruskal–Wallis test followed by Dunn’s post-test. Results are expressed as mean ± standard error of the mean (SEM).Conditions are as following: Metformin: 0.5 mM metformin hydrochloride; mix miR: mixture of miRNAs (miR-23b and miR-145 at 60 nM each); mix miR + Metformin: 0.5 mM metformin plus the mixture of miRNAs.

In the invasion assay, we found that combined therapy significantly reduces invasive capacity in both cell lines. In A2780, the co-administration of mix miR and metformin decreased the number of invading cells by 40% compared to the control (Figure 4B). Similarly, in the OV90 line, the combined treatment induced a 39% reduction (Figure 4D). These findings demonstrate that the combined administration of these molecules effectively limits in vitro invasive potential, suggesting a relevant impact on controlling cell dissemination.

### 3.4. Metformin Reduces VEGF Secretion in A2780 Spheroids

**Figure 5:**
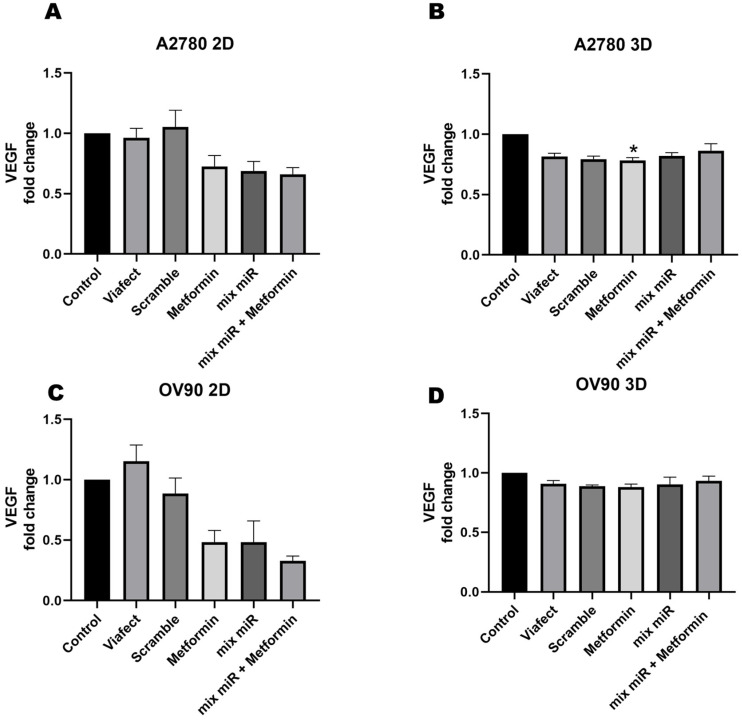
Effect of metformin and metformin plus a mixture of miRs on VEGF protein level in EOC cells. Cells were treated with metformin (0.5 mM for the 2D model and 1 mM for the 3D model), a mixture of miR (60 nM for the 2D model and 240 nM for the 3D model), and metformin plus the mixture of miRs for 48 h. An ELISA immunoassay was performed. (**A**,**B**) represents A2780 cells. (**C**,**D**) represents OV90 cells. Data were normalized with respect to Control condition and are presented as fold change. *n* = 3 independent experiments. * = *p* < 0.05, according to the Kruskal–Wallis test followed by Dunn’s post-test. Results are expressed as mean ± standard error of the mean (SEM).

VEGF quantification by ELISA in 2D and 3D models showed no significant variations across most experimental conditions for both cell lines. However, in the A2780 spheroid (3D) model, metformin treatment resulted in a 21% reduction in secretion levels compared to the control (Figure 5B). Conversely, while these data show a statistically significant, although modest, 21% decrease in VEGF levels specifically in the A2780 3D context, this observation represents a preliminary trend. Further functional validations are required to determine if this reduction translates into a definitive modulation of angiogenesis.

### 3.5. Transient Co-Transfection of miR-145 and miR-23b in Combination with Metformin Decreases Relative Area and Increases Cytotoxicity in A2780 Spheroids

**Figure 6:**
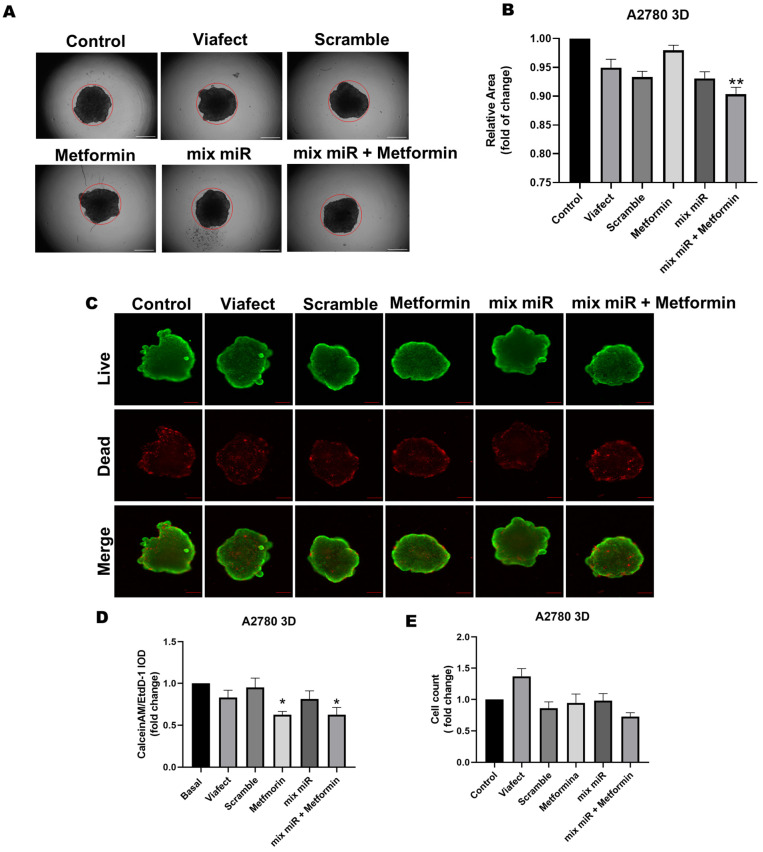
Effect of metformin plus a mixture of miRs in A2780 spheroids. (**A**) Microphotographs of A2780 spheroids treated with different conditions after 48 h. (**B**) Analysis of spheroid relative area after 48 h of co-transfection. The red circle corresponds to the control area. (**C**) Live/Dead staining images of A2780 spheroids. (**D**) Live/Dead ratio for spheroids (**E**) Spheroid cell count analysis after 48 h. All results were normalized to the Control condition. *n* = 3 independent experiments, * = *p* < 0.05; ** = *p* < 0.01 compared to the Control condition (Kruskal–Wallis test and Dunn’s post-test). Results are expressed as standard error of the mean (SEM). Scale bars represent 100 µm.

**Figure 7:**
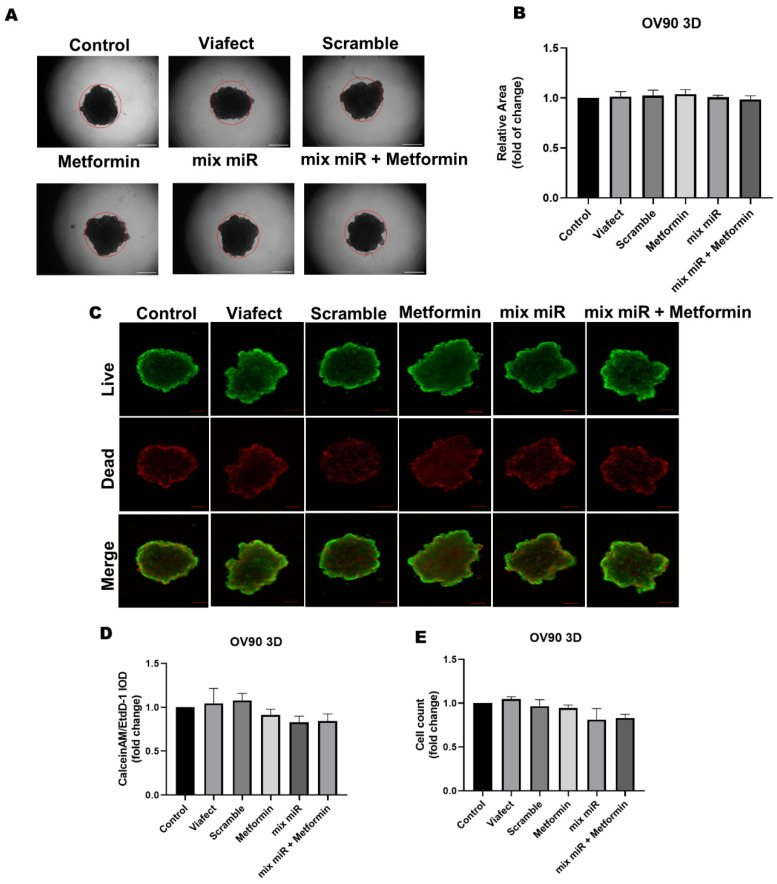
Effect of metformin plus a mixture of miRs in OV90 spheroids. (**A**) Microphotographs of OV90 spheroids treated with different conditions after 48 h. (**B**) Analysis of the spheroid relative area after 48 h of co-transfection. The red circle corresponds to the control area. (**C**) Live/Dead staining images of OV90 spheroids. (**D**) Live/Dead ratio for spheroids (**E**) Spheroid cell count analysis after 48 h. All results were normalized to the Control condition. *n* = 3 independent experiments, compared to the Control condition (Kruskal–Wallis test and Dunn’s post-test). Results are expressed as standard error of the mean (SEM). Scale bars represent 100 µm.

The evaluation in A2780 spheroids revealed that the combined therapy significantly affects the 3D structure, reducing the spheroid’s relative area by 10% (Figure 6B). Furthermore, viability analysis using the Calcein-AM/EthD-1 ratio demonstrated a marked cytotoxic effect in the 3D model. Notably, a 37% decrease in the live/dead cell proportion was observed for both the metformin treatment and the combined therapy (Figure 6D). This indicates that the addition of the miRNAs did not induce further toxicity beyond the cytotoxic effect mediated by metformin alone in this specific assay. These findings confirm that the treatments compromise cell integrity and survival within a 3D context. No effects in area, cell number, and viability were noted in OV90 3D cell cultures (Figure 7).

### 3.6. Functional Validation of miRNA Delivery in 3D Spheroids

**Figure 8:**
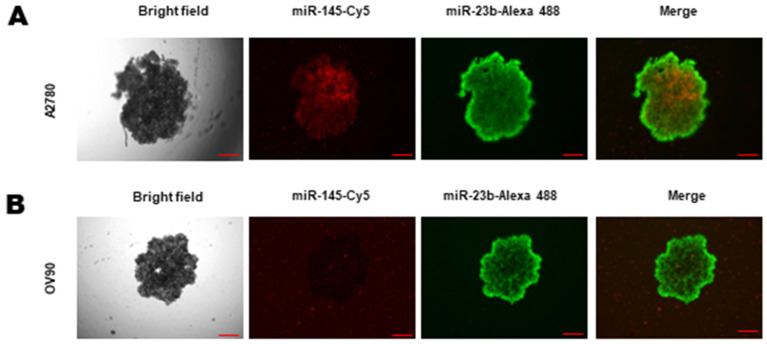
Functional validation of miRNA delivery and penetration in EOC 3D spheroids. A2780 and OV90 spheroids were co-transfected with fluorescently labeled mimics for miR-145 (Cy5, red signal) and miR-23b (Alexa 488, green signal) using ViaFect as the vehicle. After 4 h of transfection, whole-spheroid fluorescence imaging was performed to evaluate intracellular delivery. (**A**) Representative images of A2780 spheroids demonstrating successful penetration and interior distribution of both miR-145-Cy5 and miR-23b-Alexa 488 within the 3D structure. (**B**) Representative images of OV90 spheroids confirming the successful internal penetration of miR-23b-Alexa 488, while the miR-145-Cy5 signal exhibited detection limitations within this structure. Columns from left to right show: Bright Field, miR-145-Cy5 (red), miR-23b-Alexa 488 (green), and Merged images. Scale bars represent 100 µm.

To evaluate whether the differential treatment response in 3D models was related to delivery efficiency, we performed co-transfection assays using fluorescently labeled miR-145 (Cy5, red) and miR-23b (Alexa 488, green) mimics. Whole-spheroid fluorescence imaging confirmed the successful penetration of the miRNAs into the interior regions of the spheroids. In A2780 spheroids, both fluorescent signals were clearly detected throughout the intracellular space (Figure 8A). In OV90 spheroids, the miR-23b-Alexa 488 signal also successfully penetrated and distributed evenly across the spheroid interior (Figure 8B). While the Cy5-labeled miR-145 presented technical limitations regarding signal detection within the OV90 architecture, the effective internalization of the Alexa 488 signal in both models provides clear evidence that the transfection vehicle successfully delivers small RNAs into the core of these complex 3D structures.

## 4. Discussion

The dysregulation of microRNAs (miRNAs) in epithelial ovarian cancer (EOC), particularly the suppression of tumor-suppressive miRNAs such as miR-145 and miR-23b under the influence of the nerve growth factor (NGF)/TRKA axis, directly contributes to enhanced proliferation, migration, invasion, and angiogenesis. This is the established description of molecular pathogenesis in EOC [7,22]. Our results demonstrate that the co-transfection of miR-145-5p and miR-23b-3p significantly reduces Ki-67-positive cells and c-MYC expression in A2780 and OV90 lines. These findings corroborate antiproliferative mechanisms outlined in preclinical models, where miR-23b downregulated CCNG1 and miR-145-5p has been shown to target cell cycle regulators such as cyclin D2 (CCND2) and E2F transcription factor 3 (E2F3), as well as SMAD4 [23,24,25]. Furthermore, we observed a reduction in migration and in invasion. These data extend prior findings regarding the miR-145 and miR-23b role in suppressing epithelial–mesenchymal transition (EMT), as previously reported [8,9,24,25,26]. This also reinforces established evidence on both miRNA-mediated and metformin inhibition of EMT and metastasis [13,27,28,29,30].

While previous studies have demonstrated that metformin can endogenously increase the expression of tumor-suppressor miRNAs such as miR-145 and miR-23b, this effect is observed at high concentrations of metformin (10 mM) [16]. In the current study, we deliberately utilized reduced concentrations of metformin (0.5 mM for 2D cultures and 1 mM for spheroids) to approach more clinically relevant doses. At these concentrations, relying only on metformin administration to restore these crucial tumor suppressors is insufficient, particularly in chemoresistant phenotypes, such as OV90 cells [31]. Thus, our approach in this work is to achieve supraphysiological accumulation of miR-145 and miR-23b necessary to silence oncogenic targets, functioning alongside metformin’s broader metabolic disruptions to effectively limit tumor progression.

We observed a modest reduction in VEGF secretion exclusively in A2780 spheroids following metformin treatment. While this localized decrease aligns with metformin’s known capacity to reduce VEGF levels [16], this single-protein quantification is not enough to claim a definitive modulation of the complex angiogenic process. Rather, this 21% reduction represents an encouraging preliminary trend that highlights the influence of the 3D context on secretory profiles. Future studies incorporating functional angiogenic evaluations, such as in vitro endothelial tube formation, will be required to fully evaluate the physiological impact of this combined therapy on tumor-driven angiogenesis. Other mechanisms by which this drug may decrease VEGF levels may also include the downregulation of epigenetic modulators. For instance, metformin has been shown to repress histone H3 lysine 27 trimethylation (H3K27me3) and components of the polycomb repressive complex 2 (PRC2) [32]. These epigenetic modulations may affect VEGF levels as the inhibition of this complex has been shown to decrease this protein expression in other models [33,34]. In addition, it has been shown that metformin may counteract hypoxia-induced VEGF via HIF-α signaling, with reports that metformin reverses bevacizumab-induced hypoxia and attenuates stemness in the tumor microenvironment in EOC models [35]. These results underscore the limitations of 2D models in capturing EOC’s histopathological fidelity [36,37] as evidenced by studies showing differential gene expression profiles, such as upregulated hypoxia pathways in spheroids compared to monolayers [38].

Interestingly, while the combined therapy successfully reduced the overall relative spheroid area in the A2780 3D model, the viability assays revealed that the observed acute cytotoxicity was primarily induced by metformin. The co-transfection of miRNAs did not exert an additive cytotoxic effect beyond the 37% reduction achieved by metformin alone. This suggests that while the restoration of miR-145 and miR-23b contributes significantly to reduction in spheroid area and the inhibition of motility/invasion in 2D cultures, the direct cell death observed in this specific 3D context is probably a metformin-mediated metabolic response.

The contrasting responses between the 3D models underscore the biological differences not captured by traditional 2D cultures. While A2780 spheroids demonstrated sensitivity to the combined treatment, OV90 spheroids exhibited marked resistance. As it has been discussed before, greater concentrations of metformin (10 to 20-fold) show changes in viability and growth in OV90 2D and 3D models [39,40]. Regarding the transfection of our miR mix, extracting sufficient high-quality RNA from compact 500 um spheroids for precise miRNA quantification via RT-qPCR presents significant technical challenges. While our 2D transfection efficiency is supported by previously standardized and validated protocols from our group [8], to functionally validate delivery within the 3D context, we utilized fluorescently labeled miRNAs. Notably, our whole-spheroid imaging demonstrated that the lipid-based delivery vehicle successfully penetrated both A2780 and OV90 3D architecture, as evidenced by the interior distribution of miR-23b-Alexa 488. Therefore, our results suggest that the phenotypic resistance observed in OV90 spheroids is not a delivery artifact caused by the limited penetration of lipid-based vehicles, which frequently exhibit low efficiency in 3D models [41]. Instead of a purely physical barrier, this points to an intrinsic biological resistance conferred by the 3D microenvironment. Even when the miRNA successfully reaches the intracellular space, the complex extracellular matrix and altered signaling networks inherent to the 3D architecture appear to neutralize its functional impact, faithfully mimicking the intrinsic drug resistance challenges of the in vivo tumor microenvironment. This data align with preclinical paradigms where spheroids are enriched for CSCs and so exhibit enhanced chemoresistance [42,43]. Also, our in vitro combined effect aligns with epidemiological correlations showing that metformin use is associated with reduced EOC incidence and improved survival in diabetic cohorts [10,12,44]; however, clinical efficacy remains a significant challenge. A prominent example is the recent phase II clinical trial NCT02122185, which evaluated adding metformin to standard chemotherapy for non-diabetic patients with advanced EOC. Despite strong preclinical rationale, the trial yielded negative results for progression-free and overall survival [45]. This highlights how the complex tumor microenvironment and in vivo compensatory mechanisms can override promising in vitro interventions. This may suggest that administering metformin alone is likely insufficient and reinforces the necessity of a complementary strategy alongside this drug that may overload the compensatory mechanisms that cause single-agent therapies to fail.

These findings support miRNA–metformin combinations as a complementary strategy to exploit metabolic and genetic vulnerabilities in EOC, potentially overcoming resistance mechanisms inherent to different subtypes.

This study establishes the combination of miR-145/miR-23b and metformin as an inhibitor of EOC malignancy, but translational hurdles persist. Effective delivery remains a challenge, although recent advances in peptide-targeted gold nanoplatforms and chitosan encapsulation have shown promise in enhancing miRNA stability [9,46]. While our findings in 3D spheroids offer a more physiologically relevant context than traditional 2D monolayers, inherent limitations of utilizing established cell lines, such as long-term maintenance of cell lines can lead to accumulated genetic variability that may alter their intrinsic properties and influence experimental reproducibility [47]. Furthermore, a formal limitation of our in vitro study is the small sample size (*n* = 3 independent biological replicates), which necessitates preclinical validation. Additionally, monoculture spheroids lack the complex cellular diversity and stromal interactions characteristic of the in vivo tumor microenvironment [48]. Nevertheless, these standardized models remain an essential intermediate step for optimizing complex preclinical protocols like co-transfection. To address these limitations and overcome the translational gap, future investigations must rigorously validate these miRNA–metformin complementary effects utilizing patient-derived organoids (PDOs) and patient-derived xenografts (PDXs), which more accurately capture patient-specific tumor heterogeneity [18,19,37].

Additionally, addressing metformin’s AMPK-independent effects, such as the modulation of apoptosis and cell cycle arrest, could further refine this therapeutic approach [49,50]. In summary, these findings provide a preclinical rationale for miRNA–metformin complementary therapeutics, suggesting that the simultaneous inhibition of critical cellular processes and metabolic pathways could serve as a foundational strategy to eventually overcome resistance in EOC management.

## 5. Conclusions

In summary, metformin treatment combined with miR-145/miR-23b co-transfection effectively inhibits proliferation, migration, and invasion in 2D EOC models. In 3D cultures, the therapy demonstrates a complementary dynamic in sensitive models like A2780 cells, where metformin primarily mediates acute cytotoxicity, while miRNA co-transfection reduces the overall spheroid area. The fluorescence tracking revealed that the marked resistance observed in OV90 spheroids occurs despite the successful intracellular delivery of miRNA. This indicates that once the miRNA reaches the intracellular space, the altered signaling landscape of the 3D architecture can neutralize its functional effect, a phenomenon that standard 2D models fail to capture. Overall, these findings provide a solid preclinical rationale for miRNA–metformin complementary therapeutics, while strongly underscoring the absolute requirement of utilizing complex 3D models to accurately evaluate RNA-based treatments and uncover intrinsic resistance mechanisms.

## Figures and Tables

**Table 1:** miR-145 and miR-23b duplex sequences utilized for transient transfection assays.

miR	Sense	Antisense
145	5′-GUCCAGUUUUCCCAGGAAUCCCU-3′	5′-AGGGAUUCCUGGGAAAACUGGAC-3′
23-b	5′-AUCACAUUGCCAGGGAUUACC-3′	5′-GGUAAUCCCUGGCAAUGUGAU-3′

## Data Availability

The original contributions presented in this study are included in the article. Further inquiries can be directed to the corresponding author.

## Abbreviations

miRs/miRNAs	microRNAs
OC	ovarian cancer
EOC	epithelial ovarian cancer
VEGF	Vascular endothelial growth factor
